# Evaluating Patient Interest in Orthopedic Telehealth Services Beyond the COVID-19 Pandemic

**DOI:** 10.7759/cureus.16523

**Published:** 2021-07-20

**Authors:** Tyler W Henry, Daniel Fletcher, Alexander R Vaccaro, Pedro K Beredjiklian

**Affiliations:** 1 Orthopaedic Surgery, Rothman Orthopaedic Institute, Philadelphia, USA; 2 Division of Hand Surgery, Rothman Orthopaedic Institute, Philadelphia, USA

**Keywords:** telehealth, orthopedic surgery, patient, interest, telemedicine

## Abstract

Background

Patient interest and demand may have an impact on dictating the scope of orthopedic telehealth utilization beyond the coronavirus disease 2019 (COVID-19) pandemic. The purpose of this study was to assess whether current interest in orthopedic telehealth services is higher than pre-pandemic levels. Specific trends in interest, subspecialty differences, and regional differences were secondarily assessed.

Methodology

A Google Trends search was performed to assess orthopedic telehealth search interest over the last five years using the terms “Orthopedic surgeon/doctor/injury/pain + Telehealth” as well as subspecialty-specific terms. The results were formulated into combined search interest values (CSIVs), with a maximum possible value of 400, and compared between the pre-pandemic period, pre-vaccine period during the pandemic, and post-vaccine period.

Results

The pre-pandemic period mean CSIV was 40.3 (SD = 6.3), compared to 134.7 (SD = 72.1) during the pre-vaccine period, and 96.3 (SD = 4.4) during the post-vaccine period (p < 0.001). There was a positive correlation between CSIV and time (increasing weeks) during the pre-pandemic period (r_s_ = .77, p < 0.001) and no significant correlation between CSIV and time during the post-vaccine period (r_s_ = -.12, p = 0.610). Using the slope of the interest line during the post-vaccine period (y = 97.06 - 0.08x) it would take an additional 13.3 years beyond the study period to reach the mean pre-pandemic CSIV level of 40.3. Hand surgery was the subspecialty with the highest mean CSIV over the study period and general search interest was highest in Northeastern and Southeastern states during the post-vaccine period.

Conclusions

Orthopedic telehealth interest was growing before the COVID-19 pandemic and remains significantly elevated beyond pre-pandemic levels despite the reopening of clinical offices and vaccine availability across the country. It appears that a subset of patients will continue to seek telehealth services beyond the pandemic.

## Introduction

In March of 2020, the rapidly developing coronavirus disease 2019 (COVID-19) pandemic necessitated a sizable shift to telehealth platforms for orthopedic clinical visits across the United States [1,2]. The move was supported by the Centers for Medicare and Medicaid Services (CMS) expanding insurance reimbursement to mirror traditional in-person visits [3]. This insurance coverage parity eliminated a longstanding barrier to the broader incorporation of telehealth within orthopedic practices [1,4]. Prior to the pandemic, there were a small number of early investigations into the efficacy of telehealth across a myriad of orthopedic clinical scenarios [5-7]. However, technological and patient privacy concerns, insurance coverage concerns, and provider hesitation were among the factors limiting its widespread use and popularity [8]. As telehealth use increased in response to the pandemic, providers across the country innovated strategies to ensure high-quality care across virtual platforms [9].

With the widespread institution of reliable COVID-19 testing, the development of vaccines, and the decreased disease incidence and mortality rates, societal restrictions have substantially declined [10]. As a result, there has begun a progressive re-opening of clinical offices for patient visits. Telehealth volume has waned [11], bringing into question whether this method of patient interaction was a temporary measure or whether its incorporation paved the way for sustained patient interest beyond the pandemic. If patient interest in telehealth services persists above pre-pandemic levels, it will benefit orthopedic providers to continue offering the option for virtual visits, primarily driven by patient interest and demand.

Several investigators have used the Google Trends search data tool to assess patient interest in health topics and medical conditions and predicting behavior during a disease outbreak [12-16]. This tool produces normalized values reflecting interest in a given search term across a specified timeframe and location. With a five-year search across the United States, the resultant trendline includes a search interest value (SIV) between zero and 100 for each week within the past five years. Considering the impact that continued patient interest may have on dictating the scope of telehealth utilization beyond the COVID-19 pandemic [17], the purpose of this study was to assess whether current interest in orthopedic telehealth services is higher than pre-pandemic levels. Specific trends in interest, subspecialty differences, and regional differences were secondarily assessed. We hypothesized that current interest would be significantly higher than pre-pandemic levels.

## Materials and methods

A Google Trends search was performed to first assess orthopedic telehealth search interest over the last five years. This timeframe was selected in order to maximize the power and duration of analysis while producing weekly SIVs. The maximum SIV of 100 is assigned to the week during which search interest was highest, referred to here as the interest spike. The remainder of SIVs represent the weekly interest relative to that term’s interest spike. This means that rather than comparing the interest in “search term A” to all other searches over a five-year period, the data compares “search term A” to its own peak popularity over five years. For example, an average SIV of 50 before or after the interest spike would mean that search interest was approximately half of what it was at its maximum point.

Preliminary searches and correlations of the resultant SIVs were performed to determine any significant differences between similar search terms that could confound statistical analysis. This produced strong positive correlations considered to demonstrate equivalence between the terms “Surgeon” and “Surgery” (rs = .86, p < 0.001), “Orthopedic” and “Orthopaedic” (rs = .84, p < 0.001), and “Telehealth” and “Telemedicine” (rs = .89, p < 0.001). The base search term of “Orthopedic Surgeon + Telehealth” was thus developed. To fully capture patient interest, related search terms were also added, including “Orthopedic Doctor + Telehealth”, “Orthopedic Injury + Telehealth”, and “Orthopedic Pain + Telehealth”. The SIVs produced from these four searches were combined (and termed the CSIV) to represent overall patient interest in orthopedic telehealth services during the study period. The overall trendline of CSIV over the five-year period was produced and mean CSIVs were compared between the pre-pandemic period (May 29th, 2016 - March 1st, 2020), pre-vaccine period during the pandemic (March 1st, 2020 - January 3rd, 2021), and post-vaccine period (January 3rd, 2021 - May 23rd, 2021). Correlations and scatter plot line equations were used to assess the growth of telehealth interest over time during the pre-pandemic and post-vaccine periods. The weekly CSIVs were also correlated to the number of new COVID-19 cases, obtained through the Centers for Disease Control and Prevention data [18].

Next, to compare interest in telehealth services across orthopedic subspecialties, searches were performed using “Spine/Shoulder/Hand/Hip/Knee/Ankle/Foot/Sports Surgeon + Telehealth” and the mean SIVs were compared over the five-year period and among the pre-pandemic, pre-vaccine, and post-vaccine periods. Because the interest spike was assumed to be a statistical outlier higher than all other time periods, the remainder of relative SIVs were used as a marker of general interest. For example, a subspecialty with a five-year mean SIV of five would indicate less popularity than a subspecialty with a mean SIV of ten because of its lower overall interest compared to the peak pandemic interest spike.

Regional differences were assessed using the four CSIV search terms during the post-vaccine period, with the results representing comparative interest across the 50 United States and Washington D.C. These CSIVs were also correlated to the percentage of each state’s population residing in rural areas using 2010 United States Census Data [19].

Data was tracked and analyzed using the Statistical Package for the Social Sciences (SPSS Inc, Ver 26.0). Nonparametric mean testing and Spearman’s correlations were used for all analyses. Statistical significance was maintained at p < 0.05.

## Results

**Figure 1:**
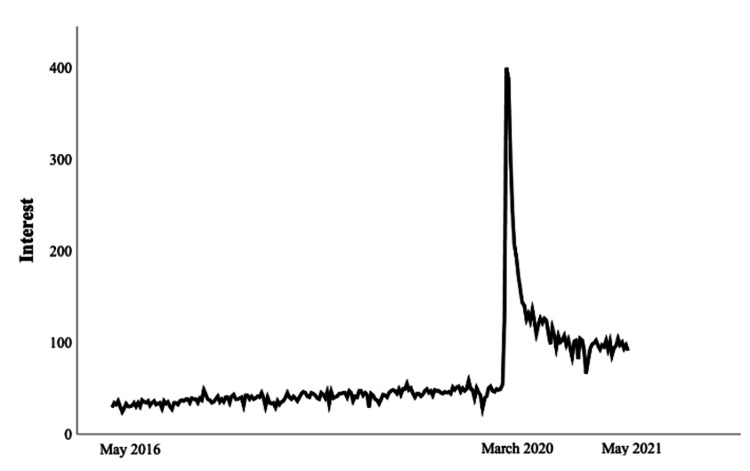
Trendline of interest in orthopedic telehealth between May 2016 and May 2021. Note: Interest is displayed in combined search interest values using Google Trends.

**Figure 2:**
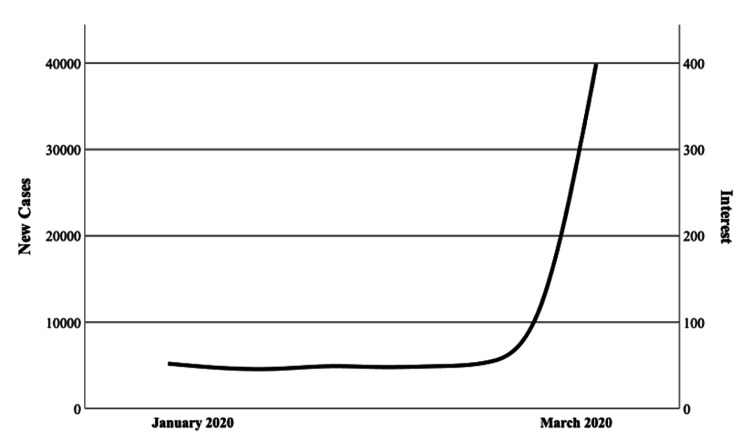
Trendline of interest in orthopedic telehealth (displayed in combined search interest values using Google Trends) compared to the number of new COVID-19 cases per week between January and March of 2020. Spearman’s correlation coefficient = 0.71 (p = 0.032).

**Figure 3:**
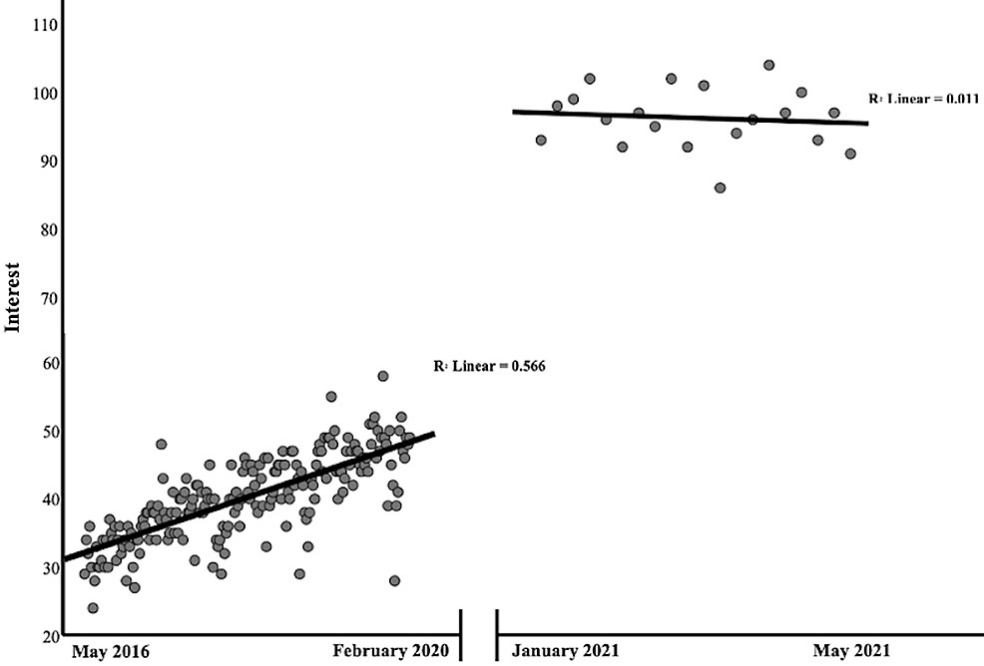
Simple scatter plot with fit lines of interest in orthopedic telehealth (combined search interest values using Google Trends) and time before the COVID-19 pandemic (increasing number of weeks between May 2016 and February 2020) and after COVID-19 vaccine development (increasing number of weeks between January 2021 and May 2021).

**Figure 4:**
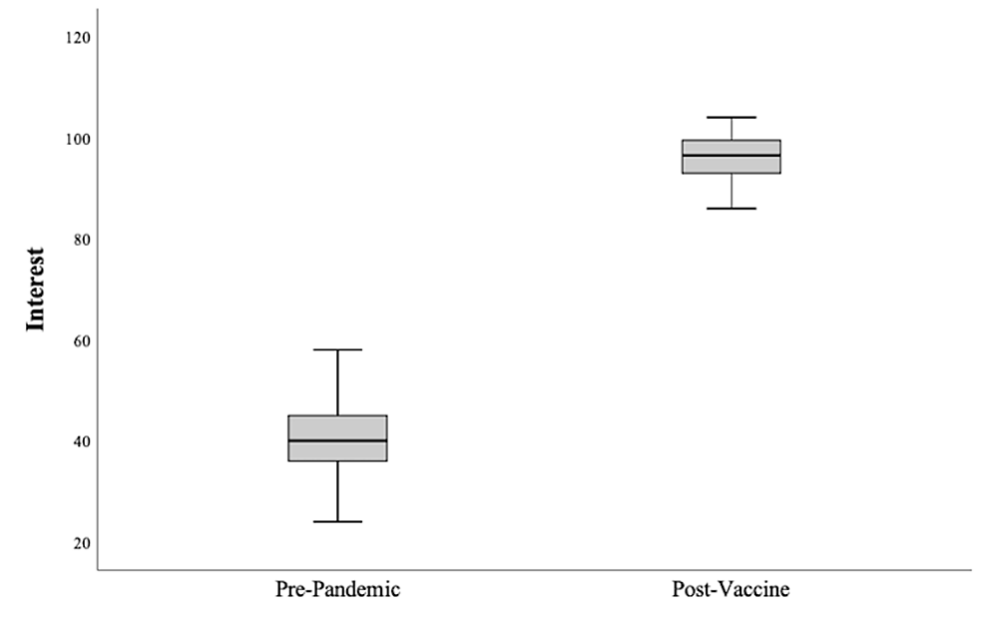
Interest in orthopedic telehealth stratified by time relative to the COVID-19 pandemic onset and the development and authorization of COVID-19 vaccinations. Note: Interest is displayed in combined search interest values using Google Trends.

Throughout the study period, peak interest in orthopedic telehealth occurred during the week of March 15th, 2020 (CSIV = 399 of possible 400), corresponding to the nationwide expansion of telehealth reimbursement by CMS on March 17th at the onset of the COVID-19 pandemic (Figure 1). From the first recorded case of COVID-19 in the United States (January 22nd, 2020), there was a strong, positive correlation between the number of new cases per week and the weekly CSIV (rs = 0.71, p = 0.032) (Figure 2). The pre-pandemic period mean CSIV was 40.3 (SD = 6.3), compared to 134.7 (SD = 72.1) during the pre-vaccine period, and 96.3 (SD = 4.4) during the post-vaccine period (p < .001). There was a positive correlation between CSIV and time (increasing weeks) during the pre-pandemic period (rs = .77, p < .001) and no significant correlation between CSIV and time during the post-vaccine period (rs = -0.12, p = 0.610) (Figure 3). Compared in isolation to pre-pandemic levels, mean CSIV during the post-vaccine period remained significantly elevated (96.3 versus 40.3, p < 0.001) (Figure 4). Using the slope of the interest line during the post-vaccine period (y = 97.06 - 0.08x) it would take an additional 13.3 years beyond the study period to reach the mean pre-pandemic CSIV level of 40.3 assuming a linear relationship.

Between May 2016 and May 2021, telehealth interest varied slightly across subspecialties, with “Hand surgeon” having the highest mean SIV (12.0; SD = 12.0) (Table 1). Significant differences in interest across subspecialties were maintained during the pre-pandemic and post-vaccine periods (Table 2).

**Table 1:** Subspecialty differences in orthopedic telehealth interest (displayed in search interest values using Google Trends) between May 2016 and May 2021. Note: Level of significance between differences is < 0.001 with Kruskal-Wallis testing.

Search term	Mean search interest value	Standard deviation
Hand Surgeon	12.0	12.0
Spine Surgeon	10.6	12.2
Knee Surgeon	10.4	12.6
Foot Surgeon	10.1	12.5
Sports Surgeon	9.9	12.6
Shoulder Surgeon	9.8	12.5
Hip Surgeon	9.7	12.3
Ankle Surgeon	9.5	12.2

**Table 2:** Subspecialty differences in orthopedic telehealth interest (displayed in search interest values using Google Trends) stratified by time period. *Kruskal-Wallis testing.

	Mean search interest values								
Time period	Hand Surgeon	Spine Surgeon	Knee Surgeon	Foot Surgeon	Sports Surgeon	Shoulder Surgeon	Hip Surgeon	Ankle Surgeon	p-value*
Pre-pandemic (5/2016 – 3/2020)	6.9	5.4	5.1	4.8	4.6	4.5	4.5	4.4	<0.001
Pre-vaccine (3/2020 – 1/2021)	30.9	30.0	30.1	29.9	29.6	29.5	29.2	28.5	0.732
Post-vaccine (1/2021 – 5/2021)	20.4	18.5	18.9	18.0	18.2	18.1	17.8	17.7	<0.001

In terms of geographical differences, since the introduction of vaccinations, orthopedic telehealth interest has been highest in Northeastern states (mean CSIV = 241.7) compared to Southeastern (mean CSIV = 206.3), Midwestern (mean CSIV = 177.9), Western (mean CSIV = 172.3), and Southwestern (mean CSIV = 169.8) states (p = 0.003) (Table 3). There was not a significant correlation between CSIV and the percentage of each state’s population living in rural versus urban/suburban areas (rs = 0.09, p = 0.535).

**Table 3:** Combined search interest values (CSIV) obtained via Google Trends assessing interest in orthopedic telehealth stratified by state between January 1st, 2021 and May 27th, 2021.

State	CSIV	State		CSIV
District of Columbia	379	Missouri		186
Vermont	308	Minnesota		180
Kansas	280	Delaware		178
New Hampshire	279	Oklahoma		178
Maine	276	Nebraska		176
Rhode Island	251	Alabama		173
Massachusetts	244	Colorado		173
New York	228	Michigan		173
Tennessee	228	Alaska		172
West Virginia	227	Pennsylvania		168
South Dakota	226	Indiana		167
Kentucky	224	Illinois		165
Connecticut	220	Oregon		164
Virginia	219	North Carolina		162
Wyoming	210	Montana		161
Mississippi	208	Louisiana		157
Hawaii	203	Wisconsin		156
Arkansas	201	Iowa		155
New Jersey	201	Texas		154
Maryland	200	Georgia		152
Florida	199	New Mexico		152
Utah	197	California		147
Idaho	196	Washington		147
Arizona	195	Nevada		125
Ohio	188	North Dakota		83
South Carolina	188			

## Discussion

As clinical offices began reopening and COVID-19 vaccines became more widely available, the future direction of telehealth within the practice of orthopedics was unclear. Some theorized that the pandemic shift served as a widespread introduction and that telehealth would remain indefinitely present within orthopedic clinical practice [20]. However, it could just as easily be theorized that telehealth was a pandemic-specific measure, and its role would soon revert to pre-pandemic levels. A key driver of telehealth’s longevity, in addition to continued insurance reimbursement, will ultimately be patient satisfaction and demand [17]. If patients remain interested in telehealth, they will likely seek providers who offer such services over those who do not. We therefore sought to assess current patient interest in orthopedic telehealth compared to pre-pandemic levels using Google Trends search data and found that interest was growing before the pandemic, remains significantly higher than pre-pandemic levels, and appears on track to remain elevated for years to come.

The growing search interest in orthopedic telehealth in the years before the pandemic is reflected in the literature, with a growing number of studies establishing its efficacy across various clinical applications, including postoperative visits [21], remote consultations [7,22], fracture management [23-26], pediatric care [27], and rehabilitation [28], among others [29-31]. Patient satisfaction throughout such investigations before and during the pandemic has been high [32-36], giving an early indication that some degree of interest would extend into the future. Our results closely support this notion, as search interest rose steadily in the years leading up to the pandemic. After the pandemic onset and interest peak, there was a sharp decline before leveling off at a value more than double the pre-pandemic average. We suspect that the COVID-19 pandemic effectively accelerated the introduction of telehealth visits to orthopedic patients. For a period, most patients were required to participate in a virtual visit, and those who preferred the experience to traditional in-person visits remained interested while those who did not sharply reverted back to seeking in-person evaluation. The bottom line is that there is significantly more interest in orthopedic telehealth after the peak of implementation than there was before the pandemic onset. It appears warranted that orthopedic practices continue to offer telehealth services to accommodate patients who may continue to prefer virtual visits.

Hand surgery garnered the most sustained interest of the included subspecialty searches by a small margin, though it is difficult to prognosticate the causative factors producing this result. The overall clinical volume between subspecialties and the types of encountered pathologies likely contributed to an unclear extent, but regional differences as well as differences in individual provider or practice volume and scope cloud such associations. There are many investigations specific to telehealth implementation in hand surgery practice [29,37-39], but little evidence to support the presently observed interest level compared to other subspecialties.

Telehealth interest during the post-vaccine period was highest in Northeastern and Southeastern states. Interestingly, in an assessment of telehealth utilization during the COVID-19 pandemic, Parisien et al. found that orthopedic practices in the Northeastern and Southern United States were most likely to offer telehealth services compared to other regions [40]. Whether or not patient interest primarily drove the results observed by Parisien et al. or if increased availability drove the higher regional interest observed presently are both unknown. Nevertheless, there is likely a cyclical interplay between patient demand and the number of practices offering telehealth services in a given region. Further investigations into the factors driving patients to seek telehealth services could further elucidate this association.

There are limitations to our study outside of its retrospective design. It is impossible to confirm that the search interest levels reflect only patients seeking telehealth services. However, we attempted to create our search terms from the patient perspective (i.e. using surgeon instead of surgery, orthopedic instead of orthopaedic, and including doctor, injury, and pain) to address this limitation. Second, as is the case with all telehealth-related studies, there is an inherent bias in the patients actively seeking or selected for telehealth visits that limit the generalizability of our results to a small extent. Not all patient scenarios are amenable to telehealth visits regardless of interest or preference. Third, the post-vaccine period in the United States was a fluid period in which vaccine rollout was not a uniform process across the country. Vaccine availability may have influenced interest during the post-vaccine period and geographical differences. Finally, using Google Trends data may be a less accurate strategy to answer the primary study question than reporting raw data related to the number of searches throughout the study period, but feasibility prevented using the latter method.

## Conclusions

In conclusion, interest in orthopedic telehealth was steadily growing prior to the onset of the COVID-19 pandemic and remains significantly elevated beyond pre-pandemic levels despite the reopening of clinical offices and the introduction of vaccine availability across the country. It appears that a subset of patients will continue to seek telehealth services beyond the pandemic. Therefore, it will likely benefit orthopaedic providers to continue offering virtual visits when clinically appropriate and preferred by patients.
